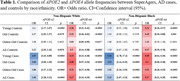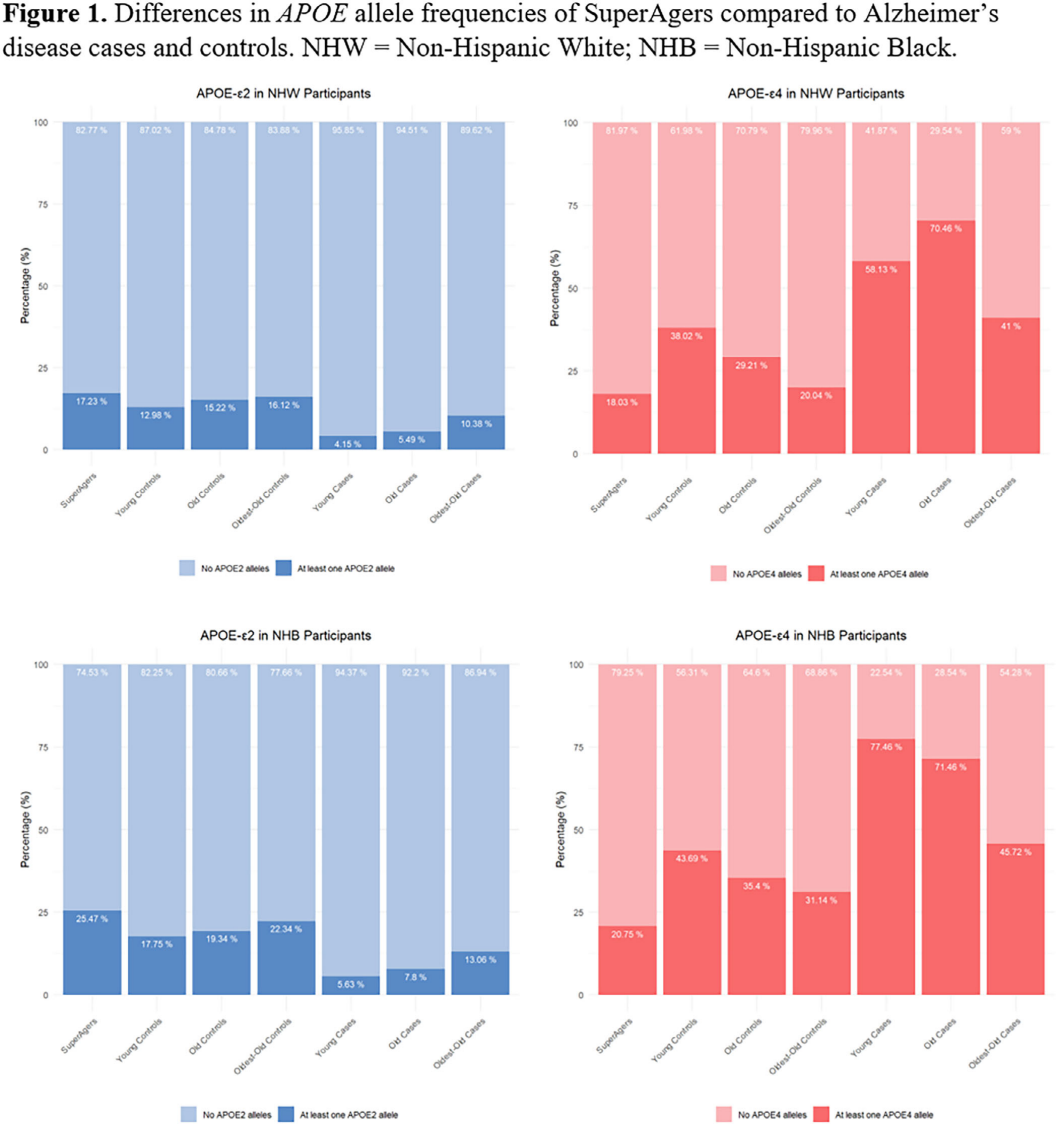# Evaluating the association between *APOE* genotypes and cognitive resilience in SuperAgers

**DOI:** 10.1002/alz.093260

**Published:** 2025-01-03

**Authors:** Alaina Durant, Shubhabrata Mukherjee, Michael L. Lee, Seo‐Eun Choi, Phoebe Scollard, Emily H. Trittschuh, Jesse Mez, William S. Bush, Brian W. Kunkle, Adam C. Naj, Katherine A. Gifford, Michael L. Cuccaro, Carlos Cruchaga, Jason J. Hassenstab, Margaret A Pericak‐Vance, Lindsay A. Farrer, Li‐San Wang, Jonathan L. Haines, Angela L. Jefferson, Walter A. Kukull, C Dirk Keene, Andrew J. Saykin, Paul M. Thompson, Eden R. Martin, David A. Bennett, Lisa L. Barnes, Julie A. Schneider, Marilyn S. Albert, Sterling C. Johnson, Corinne D. Engelman, Richard Mayeux, Badri N. Vardarajan, Paul K. Crane, Logan C. Dumitrescu, Timothy J. Hohman, Leslie S Gaynor, The Alzheimer’s Disease Neuroimaging Initiative (ADNI), Alzheimer’s Disease Genetics Consortium (ADGC), The Alzheimer’s Disease Sequencing Project (ADSP)

**Affiliations:** ^1^ Vanderbilt Memory & Alzheimer’s Center, Vanderbilt University Medical Center, Nashville, TN USA; ^2^ Department of Medicine, University of Washington School of Medicine, Seattle, WA USA; ^3^ VA Puget Sound Health Care System, Seattle Division, Seattle, WA USA; ^4^ Department of Psychiatry and Behavioral Sciences, University of Washington School of Medicine, Seattle, WA USA; ^5^ Department of Neurology, Boston University Chobanian & Avedisian School of Medicine, Boston, MA USA; ^6^ Department of Population and Quantitative Health Sciences, Case Western Reserve University, Cleveland, OH USA; ^7^ John P. Hussman Institute for Human Genomics, University of Miami Miller School of Medicine, Miami, FL USA; ^8^ Department of Biostatistics, Epidemiology, and Informatics, University of Pennsylvania Perelman School of Medicine, Philadelphia, PA USA; ^9^ Penn Neurodegeneration Genomics Center, Department of Pathology and Laboratory Medicine, Perelman School of Medicine, University of Pennsylvania, Philadelphia, PA USA; ^10^ Department of Psychiatry, Washington University School of Medicine, St. Louis, MO USA; ^11^ NeuroGenomics and Informatics Center, Washington University School of Medicine, St Louis, MO USA; ^12^ Washington University in St. Louis, St. Louis, MO USA; ^13^ Department of Medicine (Biomedical Genetics), Boston University Chobanian & Avedisian School of Medicine, Boston, MA USA; ^14^ Department of Biostatistics, Boston University School of Public Health, Boston, MA USA; ^15^ Penn Neurodegeneration Genomics Center, Dept of Pathology and Laboratory Medicine, University of Pennsylvania, Philadelphia, PA USA; ^16^ Cleveland Institute for Computational Biology, Department of Population and Quantitative Health Sciences, Case Western Reserve University, Cleveland, OH USA; ^17^ Department of Epidemiology, School of Public Health, University of Washington, Seattle, WA USA; ^18^ Department of Laboratory Medicine and Pathology, University of Washington, Seattle, WA USA; ^19^ Department of Medical and Molecular Genetics, Indiana University School of Medicine, Indianapolis, IN USA; ^20^ Center for Neuroimaging, Department of Radiology and Imaging Sciences, Indiana University School of Medicine, Indianapolis, IN USA; ^21^ Keck School of Medicine, University of Southern California, Los Angeles, CA USA; ^22^ Rush Alzheimer’s Disease Center, Rush University Medical Center, Chicago, IL USA; ^23^ Rush Alzheimer's Disease Center, Rush University Medical Center, Chicago, IL USA; ^24^ Johns Hopkins University School of Medicine, Baltimore, MD USA; ^25^ University of Wisconsin‐Madison School of Medicine and Public Health, Madison, WI USA; ^26^ Alzheimer’s Disease Research Center, University of Wisconsin School of Medicine and Public Health, Madison, WI USA; ^27^ University of Wisconsin‐Madison, School of Medicine and Public Health, Madison, WI USA; ^28^ The Taub Institute for Research on Alzheimer’s Disease and The Aging Brain, Columbia University Medical Center and The New York Presbyterian Hospital, New York, NY USA; ^29^ Taub Institute for Research on Alzheimer’s Disease and The Aging Brain, Columbia University Medical Center, New York, NY USA; ^30^ Vanderbilt Genetics Institute, Vanderbilt University Medical Center, Nashville, TN USA; ^31^ Vanderbilt Memory and Alzheimer’s Center, Institute for Medicine and Public Health, Vanderbilt University Medical Center, Nashville, TN USA; ^32^ Vanderbilt Memory & Alzheimer's Center, Vanderbilt University Medical Center, Nashville, TN USA

## Abstract

**Background:**

“SuperAgers” are older adults (ages 80+) whose cognitive performance resembles that of adults in their 50s to mid‐60s. Factors underlying their exemplary aging are underexplored in large, racially diverse cohorts. Using eight cohorts, we investigated the frequency of *APOE* genotypes in SuperAgers compared to middle‐aged and older adults.

**Method:**

Harmonized, longitudinal memory, executive function, and language scores in Non‐Hispanic White (NHW) and Non‐Hispanic Black (NHB) participants were obtained from the ADSP Phenotype Harmonization Consortium. Scores were age‐ and sex‐adjusted. SuperAgers (NHW = 1,625; NHB = 106) included individuals 80+ years of age with a memory score equal to or exceeding individuals aged 50‐64 and language and executive function domain scores within normal limits who remain cognitively normal across visits. SuperAgers were compared to Alzheimer’s disease (AD) cases (NHW = 8,400; NHB = 925) and cognitively normal controls (NHW = 7,355; NHB = 1,305), as well as age‐defined subgroups (Young = ages 50‐64, Older = ages 65‐79, Oldest‐Old = age 80+). We performed binary logistic regression analyses comparing *APOE‐*ε2 and *APOE‐*ε4 alleles (0 = none, 1 = 1+ alleles present) among SuperAgers and their counterparts, covarying for sex and education. We corrected for multiple comparisons using the Benjamini‐Hochberg procedure.

**Results:**

Across racial groups, SuperAgers had significantly higher proportions with *APOE‐*ε*2* alleles and lower proportions with *APOE*‐ε4 alleles compared to cases (Table 1, Figure 1). Similar differences were observed between SuperAgers and Young and Old Controls, although differences were restricted to *APOE*‐ε4 in NHB comparisons. NHW SuperAgers had lower proportions with *APOE*‐ε4 alleles compared to Oldest‐Old Controls; *APOE*‐ε2 proportions did not differ.

**Conclusion:**

Within our large, harmonized cohort, larger proportions of SuperAgers had *APOE‐ε2* alleles and smaller proportions had *APOE*‐ε4 alleles than AD cases across both NHW and NHB participants. Crucially, higher proportions of NHW SuperAgers had *APOE*‐ε2 alleles than younger controls (ages<80) and lower proportions had *APOE*‐ε4 alleles than all controls including age‐matched controls (ages 80+). This work provides the strongest evidence to date that APOE is associated with SuperAging. *APOE*‐ε2 did not differentiate NHB SuperAgers from controls nor *APOE*‐ε4 from other oldest‐old adults in present analyses. Future work will extend to whole genome analysis to identify novel genomic drivers of SuperAging.